# Sodium butyrate enhances the cytotoxic effect of cisplatin by abrogating the cisplatin imposed cell cycle arrest

**DOI:** 10.1186/1471-2199-11-49

**Published:** 2010-06-24

**Authors:** Miglena Koprinarova, Petya Markovska, Ivan Iliev, Boyka Anachkova, George Russev

**Affiliations:** 1 Institute of Molecular Biology, Bulgarian Academy of Sciences, Acad. G. Bonchev Street, block 21, 1113 Sofia, Bulgaria; 2 Institute of Experimental Pathology and Parasitology, Bulgarian Academy of Sciences, Acad. G. Bonchev Street, block 25, 1113 Sofia, Bulgaria

## Abstract

**Background:**

Histone deacetylase inhibitors have been proposed as potential enhancers of the cytotoxic effect of cisplatin and other anticancer drugs. Their application would permit the use of lower therapeutic doses and reduction of the adverse side effects of the drugs. However, the molecular mechanisms by which they sensitize the cells towards anticancer drugs are not known in details, which is an obstacle in developing effective therapeutic protocols.

**Results:**

In the present work, we studied the molecular mechanisms by which sodium butyrate sensitizes cancer cells towards cisplatin. HeLa cells were treated with 5 mM butyrate, with 8 μM *cis*-diaminedichloroplatinum II (cisplatin), or with both. Cells treated with both agents showed approximately two-fold increase of the mortality rate in comparison with cells treated with cisplatin only. Accordingly, the life span of albino mice transfected with Ehrlich ascites tumor was prolonged almost two-fold by treatment with cisplatin and butyrate in comparison with cisplatin alone. This showed that the observed synergism of cisplatin and butyrate was not limited to specific cell lines or *in vitro *protocols, but was also expressed *in vivo *during the process of tumor development. DNA labeling and fluorescence activated cell sorting experiments showed that cisplatin treatment inhibited DNA synthesis and arrested HeLa cells at the G1/S transition and early S phase of the cell cycle. Western blotting and chromatin immunoprecipitation revealed that this effect was accompanied with a decrease of histone H4 acetylation levels. Butyrate treatment initially reversed the effect of cisplatin by increasing the levels of histone H4 acetylation in euchromatin regions responsible for the G1/S phase transition and initiation of DNA synthesis. This abrogated the cisplatin imposed cell cycle arrest and the cells traversed S phase with damaged DNA. However, this effect was transient and continued only a few hours. The long-term effect of butyrate was a massive histone acetylation in both eu- and heterochromatin, inhibition of DNA replication and apoptosis.

**Conclusion:**

The study presents evidence that cell sensitization towards cisplatin by sodium butyrate is due to hyperacetylation of histone H4 in specific chromatin regions, which temporarily abrogates the cisplatin imposed cell cycle arrest.

## Background

Numerous reports in the recent years have described the anticancer effect of histone deacetylase inhibitors [1-3]. For the time being, it does not seem probable that they could be used in cancer therapy alone, but increasing body of evidence suggests that at least some could have a future in combination with different anticancer agents [4-6]. Sodium butyrate is the sodium salt of the butyric acid, which is a four carbon normal fatty acid and is a natural metabolite in many organisms including bacteria populating the gastrointestinal tract. Roles for butyrate have been established in a number of epigenetically controlled activities such as cell differentiation, proliferation, motility, induction of cell cycle arrest, apoptosis [7], and even in memory formation [8]. However, the mechanisms by which butyrate suppresses growth and induces cellular differentiation or apoptosis are not known in details [9]. Microarray assays of global gene expression profiles have shown that over 450 genes were significantly regulated by butyrate in bovine kidney epithelial cells. Most of them were down-regulated, but over 30 genes were up-regulated [10]. Among the down-regulated genes were genes crucial for initiation of DNA synthesis such as MCM and Orc proteins, which are essential for the assembly of the prereplication complex. CDC2/Cdk1 and related cyclins were also down-regulated. On the other hand, genes related to apoptosis were up-regulated. In another assay over 10,000 genes were found responsive to butyrate regulation in human epithelial cells [11]. Butyrate exerts several modulatory effects on nuclear proteins and DNA such as induction of histone acetylation and phosphorylation, and hypermethylation of cytosine residues in DNA [12]. The steady state of histone acetylation is controlled by the equilibrium of two distinct families of enzymes, histone acetyltransferases (HATs) and histone deacetylases (HDACs). Since the early discovery of histone acetylation by Allfrey and colleagues [13], this posttranslational modification has been correlated with the processes of chromatin assembly and transcription [14]. At present, it is well established that actively expressed genes are associated with hyperacetylated core histones, while repressed genes are associated with hypoacetylated histones [15]. Activation and repression of different genes is achieved by changes of chromatin structure. Acetylation of core histones at specific lysine residues in the NH_**2 **_-terminal tails leads to relaxation of the compact chromatin structure allowing transcriptional activators to access DNA [16]. In addition, core histones associated with DNA replication origins are hypoacetylated when the origins are inactive but undergo hyperacetylation before their firing [17,18]. Core histone acetylation and deacetylation are also associated with checkpoint activation and repression [19]. However, recent reports have suggested that the relationship of chromatin function and histone acetylation could be more complex than the simple scheme in which acetylation means activity and deacetylation means inactivity. It has been shown that not the acetylation status, but rather acetylation turnover, which could be very rapid, is important [16,20]. This might explain the results of microarray assays in which butyrate treatment, which caused global and permanent histone acetylation actually brought about repression of most of the genes assayed.

In the present paper, we have studied the sensitizing effect of the HDAC inhibitor sodium butyrate on HeLa cells towards cisplatin treatment. We have found that cisplatin arrested HeLa cells at the G1/S phase boundary and early S phase of the cell cycle and that this arrest was accompanied with reduction of histone H4 acetylation in chromatin. Butyrate treatment initially reversed the cisplatin-induced deacetylation of histone H4 in chromatin regions responsible for DNA synthesis, which led to abrogation of the cell cycle arrest. Thus by forcing the cells to traverse the S phase of the cell cycle with damaged DNA, butyrate enhanced the lethal effect of cisplatin. At later times butyrate treatment brought about massive hyperacetylation of total chromatin histone H4 and probably of all core histones and cessation of DNA synthesis.

## Results

### Synergistic effect of butyrate and cisplatin on HeLa cells death rates

**Figure 1:**
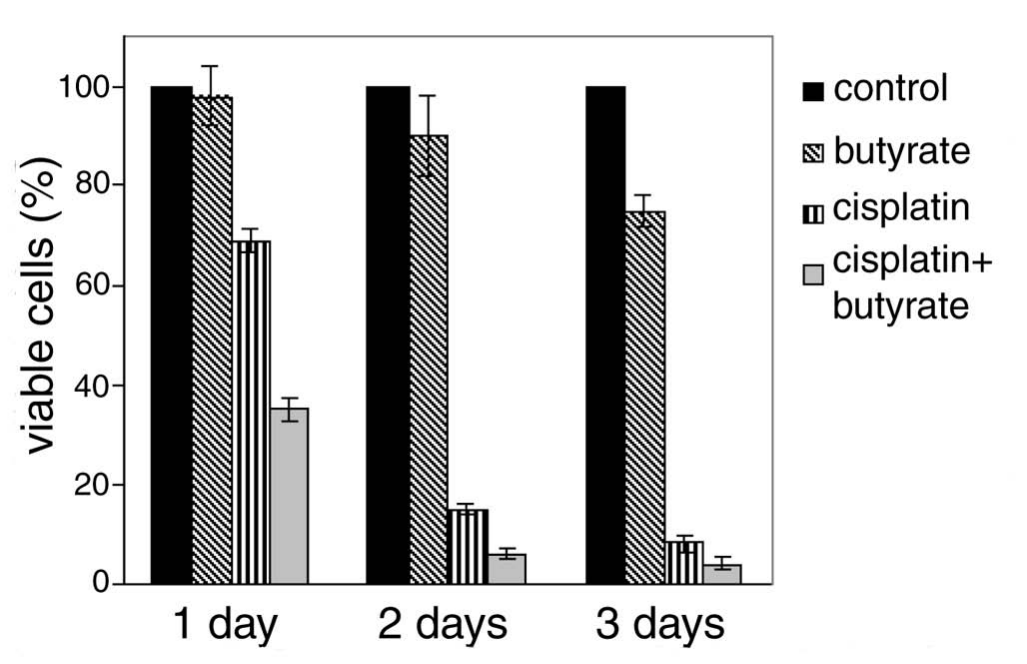
**HeLa cells mortality rates **. Exponentially growing HeLa cells were treated with 8 μM cisplatin, with 5 mM butyrate, or with both. At days 1, 2 and 3 after the treatment, cells were collected, stained with trypan blue and counted. The numbers of viable cells were expressed as percentage of the number of control cells at day 1. The results are means of three independent experiments and standard deviations are shown with error bars.

The first experiments we carried out were to establish the synergistic effect of butyrate and cisplatin on tumor cells. To follow the kinetics of cell survival we treated exponentially growing HeLa cells with 5 mM butyrate, with 8 μM cisplatin and with a combination of both agents. One, 2 and 3 days later cells were stained with trypan blue to mark the dead cells, and the viable cells were counted. The results showed that the cells treated with butyrate gradually stop to proliferate during the first day and in the course of the next 3 days about 20% of them died. This result is in agreement with the literature data that butyrate depletes cellular populations of S-phase cells and causes apoptosis [21]. Nevertheless, at day 1, almost all cells were alive and over 75% of them remained alive in the course of the following 3-day period. On the contrary, cells treated with cisplatin began to die from the very beginning in a time dependent way and at day 1 about 30% (p ≤ 0.01) of them were dead. Treatment with both agents simultaneously showed that butyrate and cisplatin had synergistic effect. In this case, over 60% (p ≤ 0.0005) of the cells were dead at day 1 (Fig.1).

### Synergistic effect of butyrate and cisplatin *in vivo*

Next, we showed that the observed synergism was not limited to a specific cell line or *in vitro *protocol, but was also expressed *in vivo *during the process of tumor development. To this end, we used Ehrlich-Lettre hyperdiploid ascites tumor (EAT). Albino mice were transfected with the tumor by an intraperitoneal injection of undiluted ascites liquid. The tumor cells rapidly proliferated in the abdominal cavity increasing the tumor volume and the animals died 10-11 days after the inoculation. This makes EAT a suitable model for rapid, preliminary assessment of the anticancer effect of different agents [22-24]. We inoculated three groups of mice, each consisting of 5 animals, with EAT. Twenty four hours later, they received 5 mg/kg cisplatin, or 166 mg/kg butyrate, or both by a single intraperitoneal injection. The time of death of each animal was recorded. The control animals died 9 to 13 days after transfection with EAT, as expected. Cisplatin treatment led to 1-2 days increase of the life span of the mice (not statistically significant). Butyrate treatment in the applied concentration prolonged the life span of the transfected animals with approximately 3 days (not statistically significant). In agreement with the results with HeLa cells, combined treatment with cisplatin and butyrate almost doubled (statistically significant) the survival period of EAT transfected mice (p = 0.0019), showing that *in vivo *butyrate also enhances the cytotoxic effect of cisplatin on tumor cells as it does *in vitro *(Table 1).

**Table 1:** Survival of mice tranfected with EAT and treated with butyrate, cisplatin and both.

Treatment	Life-span (days)*	T/C**	**P **^**#**^
Controls	10.8 ± 2.0	1	

Butyrate	14.0 ± 4.5	1.3	0.19

Cisplatin	12.0 ± 3.5	1.1	0.53

Cisplatin + Butyrate	19.0 ± 3.4	1.8	0.0019

* Albino mice were injected intraperitoneally with undiluted EAT liquid (day 0). After 24 hours (day 1) groups of five animals received another intraperitoneal injection with cisplatin, butyrate or both. Mean life spans and the standard deviations are shown.

** The ratio between the mean life spans of the treated animals (T) and the controls (C).

^# ^The probability between control and experimental groups assuming null hypothesis.

### Effect of cisplatin and butyrate on the cell cycle

**Figure 2:**
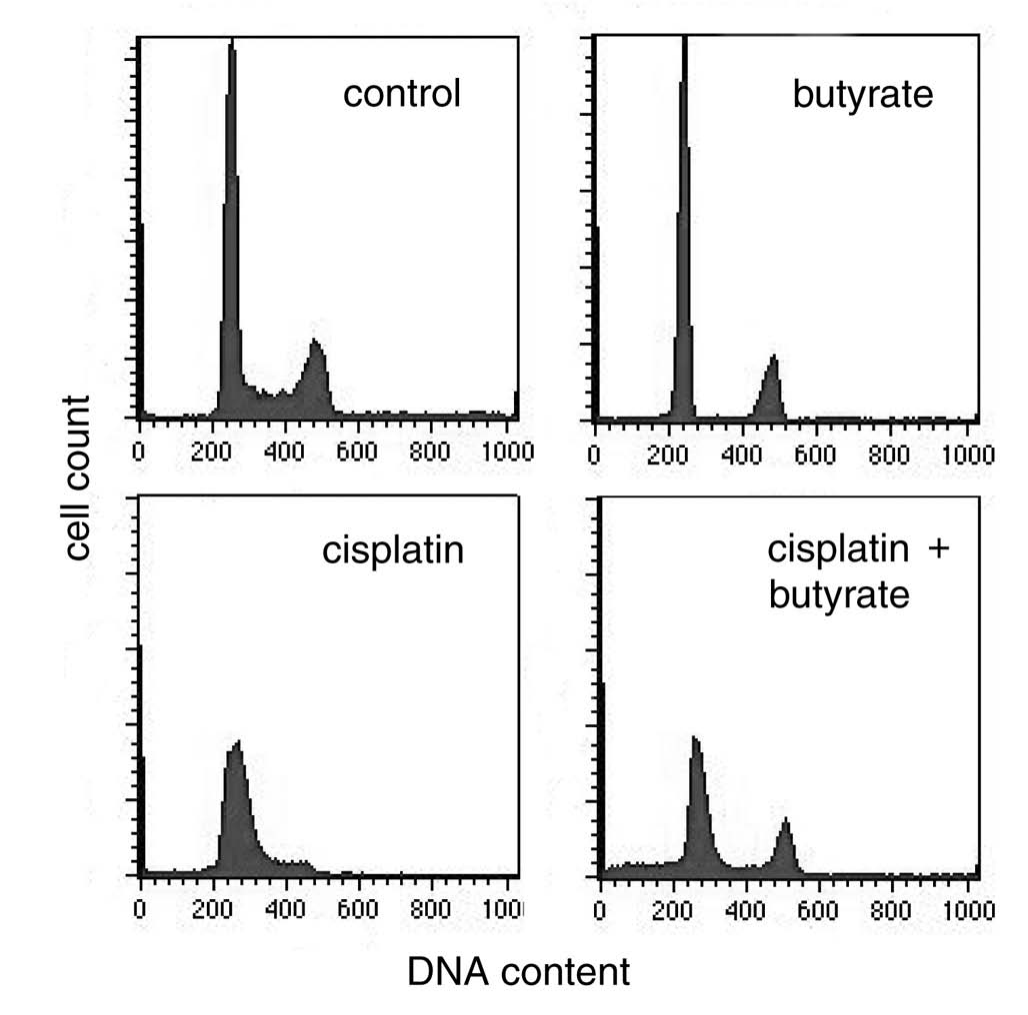
**FACS analysis of HeLa cells **. HeLa cells were treated with 8 μM cisplatin, with 5 mM butyrate, or with both. Twenty four hours after the treatment cells were fixed, stained with propidium iodide and 2 × 10^4 ^cells were subjected to FACS analysis.

After having established that butyrate enhances the cytotoxic effect of cisplatin both *in vitro *and *in vivo*, we investigated the molecular mechanisms underlying this synergism. It should be noted, that although cisplatin is widely used and for a long time, the precise mechanisms of its cytotoxicity remain unknown. Its main target is DNA where it produces mostly intrastrand bridges that are located in the major groove of the DNA double helix and block transcription and DNA replication. There are data that cisplatin treatment causes G1 and S phase cell cycle arrest, during which cells repair the cisplatin-induced damage [25,26]. Further, it was shown that retinoblastoma protein (Rb) deficiency could abrogate the cisplatin induced cell cycle arrest, the bypass resulting in increased sensitivity to the drug both in cell cultures and xenograft models [27,28]. Rb has diverse functions in the cell cycle progression and differentiation. Its major role is to recruit histone deacetylases to the E2F target genes responsible for the G1/S phase transition thus keeping them suppressed and maintaining the G1 status. In this way, histone deacetylation correlates with repression of E2F target genes and G1/S arrest, while histone acetylation is connected with S phase progression. For this reason, we presumed that effect similar to that of Rb inactivation could be achieved with HADC inhibition since the result of both treatments would be acetylation of histones in the vicinity of the E2F binding sites, abrogation of the cisplatin induced arrest and S phase transition. To check this possibility we studied the effect of cisplatin and butyrate on the cell cycle. Exponentially growing HeLa cells were treated with 8 μM cisplatin, with 5 mM sodium-butyrate, or with both for 24 hours and the cell cycle distribution was determined by flow cytometry. Our results showed that exponentially growing HeLa cells had the following cell cycle distribution: 60% in G1, 20% in S and 18% in G2/M phases. In agreement with the literature data [29,30], cells treated with butyrate for 24 hours were blocked mostly in G1 but also in G2 phases and were depleted of S-phase cells. Cells treated with cisplatin were blocked at the G1/S boundary and early S phase of the cell cycle with only 2-3% being in G2 phase. In addition, certain amount of sub-G1 phase material was present indicating that part of the cells have undergone apoptosis. The cell cycle distribution profiles of HeLa cells treated with cisplatin and butyrate differed from that of cells treated with cisplatin in two aspects. First, the amount of the sub-G1 material, indicative for apoptosis, has increased. This result confirmed the results from the survival experiments (Fig. 1) showing the synergistic effect of cisplatin and butyrate. Second, the S phase fraction was decreased while the G2 phase fraction was significantly increased to 17-18% (p ≤ 0.005), which showed that butyrate had relieved the cisplatin induced cell cycle arrest and part of the cells have traversed the S phase with damaged DNA (Fig. 2).

### Effect of cisplatin and butyrate on DNA synthesis

**Figure 3:**
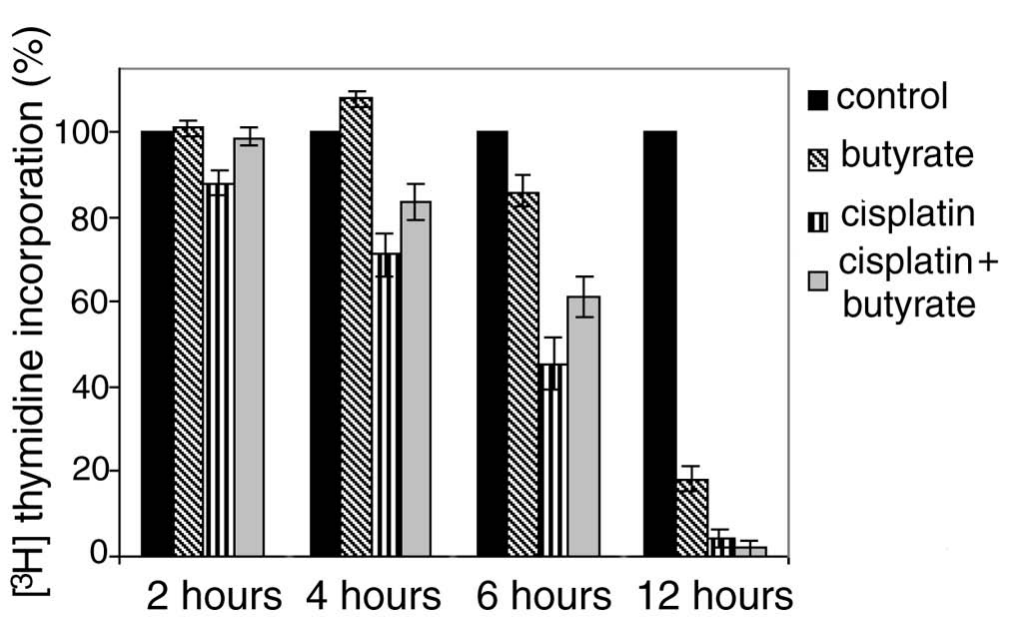
**DNA synthesis in HeLa cells **. HeLa cells were treated with 8 μM cisplatin, with 5 mM butyrate, or with both. At the specified times aliquots were withdrawn, 1 μCi/mL of ^3^H-thymidine was added and 30 min later the cells were collected, washed and counted. ^3^H incorporation was expressed as percentage of the control. The results are means of three independent experiments and standard deviations are shown with error bars.

To follow the butyrate induced S phase transition, we studied the effect of cisplatin and butyrate on DNA synthesis in the course of the first several hours after the treatment. We treated exponentially growing HeLa cells with 8 μM cispaltin, with 5 mM butyrate, or with both. At 2 hours intervals aliquots were withdrawn, 1 μCi/mL of ^3^H-thymidine was added and 30 min later, cells were collected, washed and counted. Our results showed that butyrate alone had initial positive effect on DNA synthesis. ^3^H-thymidine incorporation of HeLa cells treated with butyrate increased at the 4^th ^hour by about 10-15%, after which began to decrease. Other authors have also reported an initial increase of the S phase cell population, followed by inhibition of proliferation [21]. A possible explanation could be that due to the relaxation of chromatin structure caused by the hyperacetylation of core histones, additional replication origins were activated. On the other hand, cisplatin began to inhibit DNA synthesis almost immediately and at the 6^th ^hour the ^3^H thymidine incorporation decreased by over 50% (p ≤ 0.0025). In a previous paper, we have shown that the initial inhibition of DNA synthesis by cisplatin was a result of S phase arrest due to blocked DNA elongation of the already initiated DNA chains [31]. Nevertheless, the ^3^H thymidine incorporation continued for several hours due to the continuous entry of G1 cells into S phase and the subsequent activation of new replication origins. However, after 12 hours, DNA synthesis was completely inhibited, which showed that at this time G1 phase arrest had also been established and both elongation of the already initiated DNA strands and the initiation of new DNA strands had been blocked. The simultaneous treatment of the cells with cisplatin and butyrate had an effect that was a combination of the effects of the two agents and in this case, the ^3^H thymidine incorporation increased between the 4^th ^and the 6^th ^hours by 12-15% (p ≤ 0.11) in comparison with the cells treated with cisplatin only. A conclusion can be drawn that butyrate relieved the cisplatin imposed block on DNA synthesis and temporarily permitted the damaged cells to traverse the S phase. For this reason, the cells treated with the two agents showed higher levels of DNA synthesis than the cells treated with cisplatin only (Fig. 3). We supposed that the observed cisplatin induced arrest could be caused by deacetylation of core histones and that butyrate, which is a potent HDACs inhibitor, prevented deacetylation thus permitting the cells to enter and traverse the S phase. However, this effect was temporary and 12 hours later in all three cases, i.e. cisplatin, butyrate and cisplatin plus butyrate, DNA synthesis was inhibited (Fig. 3).

### Effect of cisplatin and butyrate on total histone H4 acetylation

**Figure 4:**
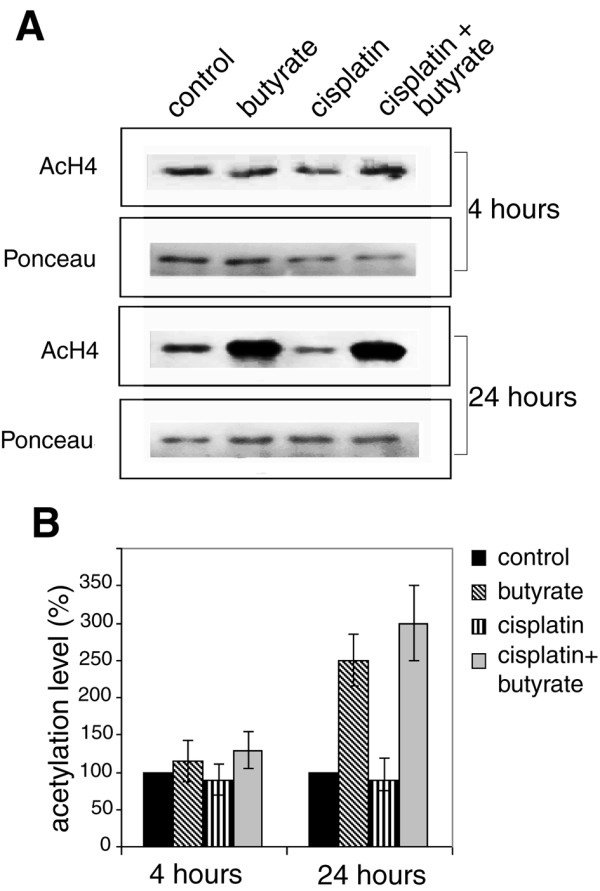
**Western blotting of acetylated histone H4 **. HeLa cells were treated with 8 μM cisplatin, with 5 mM butyrate, or with both. Four and 24 hours after the treatment aliquots were withdrawn, total histone fraction was isolated and equal amount of histones were subjected to SDS-polyacrylamide gel electrophoresis. (A) The proteins were transferred onto nitrocellulose membranes and stained with anti-acetylated histone H4 antibody. Ponceau S staining of H4 is shown as loading control. (B) The acetylated histone H4 was visualized and quantified by the Odyssey scanning system. The acetylation levels are expressed as percentage of the untreated control. The results are means of five independent experiments and standard deviations are shown by error bars.

To shed more light on the effect of cisplatin and butyrate on histone H4 acetylation we used Western blotting. Exponentially growing HeLa cells were treated with 5 mM butyrate, 8 μM cisplatin or both for 4 and 24 hours and equal amounts of histones were subjected to SDS-polyacrylamide gel electrophoresis. They were transferred onto nitrocellulose membrane and acetylated histone H4 was determined with an antibody against acetylated histone H4. Our results showed that when HeLa cell were treated with butyrate for 4 hours, total histone H4 acetylation increased by approximately 30% (p ≤ 0.16). Treatment with cisplatin decreased the acetylation level of histone H4 in comparison with the control by 30-40% (p ≤ 0.093). The combined treatment with cisplatin and butyrate increased the acetylation level of histone H4, which was an indication that butyrate overruled the cisplatin imposed block on histone H4 acetylation. When the cells were treated as above, but for 24 hours, the results were in the same direction with the only difference that the acetylation levels of histone H4 in butyrate treated cells were 2-3 times higher than in the cells treated with the respective agents for 4 h only (Fig. 4A, B). A conclusion can be drawn that butyrate treatment brings about a gradual acetylation of histone H4, which continues for many hours and probably depends on the chromatin structure. It seems plausible to assume, that upon inhibition of histone deacetylases, acetylation of histone H4 first occurs at chromatin regions with more relaxed structure such as euchromatin where HATs are already present, or have easy access. This hyperacetylation of the euchromatin regions would abrogate the cisplatin induced cell cycle arrest and was probably the reason for the observed enhancment of DNA synthesis in the first hours of butyrate treatment (Fig. 3). At later times, acetylation of histone H4 in heterochromatin also takes place and this was probably connected with the G1 and G2 phases arrest and apoptosis.

### Effect of cisplatin and butyrate on acetylation of histone H4 at specific chromatin regions

**Figure 5:**
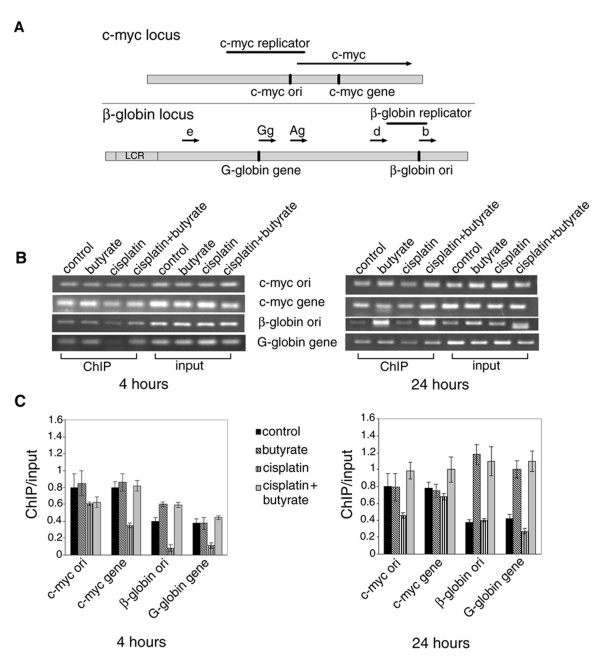
**Acetylation status of chromatin at four DNA sequences: c-myc ori, c-myc gene, β-globin ori and G-globin gene, upon treatment with cisplatin and butyrate **. (A) Map of the human c-myc and β-globin loci. The positions of the origins of replication zones (replicators) are shown by solid lines and the genes are shown by arrows. The four sequence-tagged sites (STSs) amplified by PCR are designated as c-myc ori and c-myc gene and β-globin ori and G-globin gene and shown with vertical lines. (B) HeLa cells were treated with 8 μM cisplatin, with 5 mM butyrate, or with both and 4 h or 24 h after the treatment, chromatin was immunoprecipitated with anti-acetylated histone H4 antibody. Aliquots from the immunoprecipitated (ChIP) and input (Input) DNA were subjected to PCR using primers for the respective DNA sequences. The PCR products were separated on 2.5% agarose gels and stained with ethidium bromide. (C) The DNA fractions were scanned and quantified and the ratios ChIP/Input for the four sequences after the different treatments were shown. Results are means of three independent experiments and the standard deviations are shown with error bars.

To check this hypothesis the acetylation of histone H4 at specific chromatin regions was studied by chromatin immunoprecipitation. HeLa cells were treated with butyrate, cisplatin and both for 4 and 24 hours. The cells were crosslinked with formaldehyde and sonicated on ice to obtain DNA fragments with average length of 300-500 bp. Aliquots were withdrawn for input DNA preparations and the rest of the samples were immunoprecipitated with anti-acetylated histone H4 antibody. Equal amounts of immunoprecipitated (ChIP) and control (Input) DNA were used as templates to amplify four DNA sequences. Two of the sequences were within the euchromatic c-myc region - from the early replicating c-myc origin of DNA replication and the actively transcribed c-myc gene, respectively. The other two sequences were within the nontranscribed β-globin region and were from the late replicating β-globin origin of replication and the nontranscribed globin G gene (Fig. 5A). These sequences are located in heterochromatin [17,21,32]. In agreement with our previous results [18], in control cells both the c-myc origin and the c-myc gene showed higher levels of histone H4 acetylation than the late replicating β-globin replication origin and the nontranscribed globin G gene. Butyrate treatment for 4 hours did not significantly change this picture probably because histone H4 in the vicinity of the c-myc origin and the c-myc gene was already acetylated, while the treatment period of 4 hours was not sufficient to induce acetylation of histone H4 in heterochromatin. Cisplatin treatment for 4 hours decreased histone H4 acetylation by 30% to 70% in all four chromatin regions (Fig. 5C). Since this coincided with the inhibition of the DNA synthesis, a conclusion could be drawn that the cisplatin imposed cell cycle arrest was associated with deacetylation of histone H4. This conclusion is in agreement with the experiments described in an earlier paper [19] in which we have reported that UV light induced cell cycle arrest was also accompanied with a decrease of histone H4 acetylation. When butyrate was applied simultaneously with cisplatin, it restored the acetylation levels of histone H4 to those of control cells, which led to abrogation of the cell cycle arrest, resumption of the DNA synthesis and transition through the S phase (Figs. 2, 3). When the same experiments were carried out for 24 hours, butyrate treated cells showed higher histone H4 acetylation in the heterochromatic β-globin replication origin and the nontranscribed globin G gene both in the absence and in the presence of cisplatin (Fig. 5C). This indicated that at this time hetrochromatin was also acetylated in addition to euchromatin, which was probably the reason for the observed inhibition of DNA synthesis and cell cycle arrest (Figs. 2, 3).

## Discussion

Cisplatin is an effective chemotherapeutic agent against a number of cancers such as head and neck cancer. Nevertheless, it exhibits two major drawbacks that limit its application in cancer therapy. These are its severe side effects and the rapid development of drug resistance [33]. They are mutually connected because the adverse side effects do not permit the application of high enough doses and on the other hand, under-dosing leads to development of resistance of the cancer cells. For this reason, drugs that sensitize cancer cells towards cisplatin could increase its therapeutic efficacy. Butyrate and other HDAC inhibitors have shown such potential, but we still do not know the molecular mechanisms of this sensitization, which is an obstacle in designing effective therapeutic procedures. Here we have analyzed the mechanism by which butyrate sensitizes HeLa cells towards the action of cisplatin. We examined the effects of butyrate and cisplatin on DNA replication and on the acetylation of histone H4 in eu- and heterochromatin. Our results showed that cisplatin treatment inhibited DNA synthesis and arrested HeLa cells at the G1/S transition and early S phase of the cell cycle. This effect was accompanied with hypoacetylation of the core histone H4 in both eu- and heterochromatin. On the other hand, butyrate exhibited two different effects on HeLa cells, which could be arbitrarily designated as short-term and long-term effects. The short-term effect, which occurred during the first 4-6 hours, was characterized by hyperacetylation of histone H4 and probably of other core histones in euchromatin regions associated with specific DNA sequences responsible for the G1/S phase transition and DNA replication. This effect overruled the cisplatin imposed block on DNA replication and the cells traversed the S phase with damaged DNA. Due to this effect in the early hours of its application, butyrate enhanced the cisplatin cytotoxic effect. During the second phase, butyrate caused indiscriminate hyperacetylation of core histones including those in heterochromatin, and probably other proteins, which led to inhibition of DNA synthesis, down-regulation of genes connected with cell cycle progression and triggered apoptosis. These results are in agreement with other reports showing that the effect of butyrate is time dependent. It has been shown that in the first hours after butyrate treatment cellular histone deacetylases are inhibited, core histones are hyperacetylated and many genes that have been repressed are activated. Later on irreversible changes connected with the expression of p21, Rb, Id1 and other regulatory genes take place leading to cell cycle arrest in G1 and G0, to terminal differentiation and finally to apoptosis [4,21].

The approach to sensitize cells towards the action of anticancer drugs by abrogation of the cell cycle checkpoints has already been applied - by inhibition or knock down of the checkpoint kinases Chk1, Chk2, or MK2 [34-36]. Other authors have tried to knock out, or knock down key regulatory proteins such as Rb, p53, p21, etc [27,28]. The knock out of Rb leads to a 2-fold increase of the lethal effect of cisplatin both *in vivo *and *in vitro*. Rb is a crucial player in the G1 state maintenance by preventing hyperacetylation of core histones at genes important for DNA replication. Its absence or inactivation permits their acetylation and ensures the G1/S transition. Our results are in agreement with these findings showing that the acetylation status of the core histones is important for cell cycle signaling. Thus, it seems logical to suggest that the mechanism by which butyrate, a potent HDAC inhibitor, sensitized the cells towards cisplatin was associated with hyperacetylation of core histones and abrogation of the cisplatin imposed cell cycle arrest.

The data presented here underlie both the importance of timing and the limitations of the combined application of cisplatin and butyrate in cancer treatment. Our results are in agreement with the finding that when butyrate is applied simultaneously with, or after cisplatin, the synergistic effect is stronger than when butyrate is applied first [6]. They also show that there is a specific a few hours window after butyrate administration during which it could sensitize the cells towards the action of cisplatin and that outside this window, butyrate would have little or no effect as enhancer.

## Conclusions

In this paper, we investigated the molecular mechanisms through which butyrate sensitized cells towards cisplatin. We showed that cisplatin arrests HeLa cells at the G1/S transition and early S phase, which is accompanied with reduction of histone H4 acetylation. Initially butyrate reverses this effect and by increasing histone H4 acetylation in euchromatin regions permits the cells to traverse S phase with damaged DNA. This increased the cell mortality thus enhancing the cytotoxic effect of cisplatin. Later, butyrate itself caused a G1 phase arrest and its synergistic effect decreased. This finding indicates both the importance of timing and the limitations of the combined application of cisplatin and butyrate in cancer treatment. A conclusion is drawn that i) butyrate can enhance the cytotoxic effect of cisplatin only if applied simultaneously, or shortly after it, and ii) the period during which butyrate enhances cisplatin cytotoxicity is limited to the first few hours of its application.

## Methods

### Cells and treatment

Human HeLa cells (obtained from the American Type Culture Collection) were cultured in monolayer in D-MEM with 10% foetal bovine serum supplemented with antibiotics in 5% CO_2 _atmosphere. The cell cycle distribution was determined by fluorescence activated cell sorting (FACS) analysis. The cells were washed with phosphate buffered saline (PBS), pH 7.4, fixed in 70% ethanol overnight and collected by centrifugation at 1000 × g for 10 min. They were resuspended in PBS, treated with 20 μg/mL RNase for 30 min at 37°C and stained with 20 μg/mL propidium iodide at room temperature for 90 min. 2 × 10^4 ^cells/sample were analyzed with a Becton Dickinson (Facscalibur) cell sorter, using CellQuest software (Becton Dickinson).

Fresh stock solutions of 1 mg/mL of *cis*-diaminedichloroplatinum II (cisplatin) (Sopharma, Sofia, Bulgaria) in PBS and 1 M sodium butyrate were added to the cell cultures to the desired final concentrations and the cells were further cultured for the specified periods. For labeling of DNA, ^3^H-thymidine with specific radioactivity of 37 MBq/mL (GE Healthcare, Amersham) was used. After the labeling period, cells were washed with PBS, precipitated in ice-cold 15% trichloroacetyc acid (TCA), retained on glass fiber filters (GF/C, Millipore), washed with ice-cold 5% TCA and counted with a Beckmann LS 1801 scintillation counter. Death cells were determined after staining with 1% Trypan blue for 10 min.

### Animal model

Three month-old ICR albino mice weighing 20 g were injected intraperitoneally with 0.3 mL (10^7 ^cells) undiluted ascites liquid of Ehrlich-Lettre hyperdiploid ascites tumor. 24 hours later groups of 5 animals received intraperitoneally either 5 mg/kg (100 μl of stock solution containing 1 mg/mL cisplatin in PBS) cisplatin, or 166 mg/kg (100 μl of 0.3 M stock solution of sodium butyrate in 0.14 M NaCl) sodium butyrate, or both. Mice were kept on standard laboratory diet. The time of death of each animal was recorded and the mean life spans and the standard deviations were calculated. Differences between control group and the experimental groups were estimated using Student's t test. A probability level of 0.05 was chosen for statistical significance. The experiments were performed in accordance with the guide for Care and Use of Laboratory Animals, a work permission №11130007 of the Institute of Experimental Pathology and Parasitology, Bulgarian Academy of Sciences.

### Chromatin immunoprecipitation

HeLa cells were crosslinked with formaldehyde and then sonicated with Branson sonifier cell disrupter, 70% duty cycle, 15 s pulses, 3 pulses with 1 min intervals between pulses, on ice, to obtain DNA fragments with average length of 200-500 bp. Aliquots were withdrawn for input DNA preparations and the rest of the samples were immunoprecipitated with anti-acetylated histone H4 antibody kit (Upstate Biotech) as recommended by the manufacturer. Formaldehyde crosslinks were removed at 65°C for 4 hours and DNA was isolated by phenol/chloroform extraction and ethanol precipitation.

### PCR and gel electrophoresis

The DNA sequences were amplified by 33 PCR cycles using the following primers: c-myc-ori (1829-1891) forward: CGCGCCCATTAATACCCTT, reverse: AGGGCCGCGCTTTGA; c-myc gene (4488-4552) forward: TTGTGTGCCCCGCTCC, reverse: TTCCTGTTGGTGAAGCTAA; globin-G gene (33029-133107) forward: TTTAACTTCCAAAGAACAAGTGC, reverse: GCGGCTAAAAGACCAGA; β-globin ori (62073-62147) forward: CAGGAGCAGGGAGGGCAGGA, reverse: GAAGCAAATGTAAGCAATAGATGG. The numbers in the brackets indicate the positions of the corresponding sequence-tagged sites (STS) in GenBank [17]. The PCR products were run on 2.5% agarose gels and stained with ethidium bromide. Gels were scanned and quantified with Gel-Pro Analyzer 3.1 software.

### Western blotting

Cells were washed twice with PBS, lysed in 0.5% TritonX-100 in PBS and the crude nuclear fraction recovered by centrifugation. Total histone was isolated by extraction with 0.2 N HCl for 2 hours in the cold. Protein concentrations were determined spectrophotometrically at 280 nm and 20 μg of protein of each sample were fractionated by 15% SDS-polyacrylamide gel electrophoresis. Histones were transferred to nitrocellulose Hybond-C membrane (GE Healthcare, Amersham) using Towbin transfer buffer (25 mM Tris-HCl, 192 mM glycine pH 8.6, 20% methanol, 1% SDS). After blocking in Odyssey blocking buffer (LI-COR Biosciences), the membranes were incubated with rabbit antibody to acetylated H4 (Upstate, diluted 1:2000), washed with TBS (50 mM Tris-HCl, 150 mM NaCl, pH 7.4) containing 0.01% Tween 20, incubated with Odyssey goat anti-rabbit secondary antibody (LI-COR Biosciences), washed with TBS with 0.01% Tween 20, and visualized and quantified by the Odyssey scanning system. Membranes were stained with Ponceau S to determine the amount of histone H4.

## Authors' contributions

MK carried out FACS, ChIP and DNA labeling experiments; PM carried out the Western blotting; II carried out the animal studies; BA participated in the design of the experiments and helped to draft the manuscript; GR conceived the study, and planned and supervised the experiments. All authors read and approved the final manuscript.
